# Interventions to reduce risk for sexually transmitted infections in adolescents: A meta-analysis of trials, 2008-2016

**DOI:** 10.1371/journal.pone.0199421

**Published:** 2018-06-28

**Authors:** Alexandra Morales, José P. Espada, Mireia Orgilés, Silvia Escribano, Blair T. Johnson, Marguerita Lightfoot

**Affiliations:** 1 Department of Health Psychology, Miguel Hernández University, Elche, Spain; 2 Department of Psychological Sciences, Institute for Collaboration on Health, Intervention, and Policy (InCHIP), University of Connecticut, Storrs, Connecticut, United States of America; 3 Department of Medicine, University of California, San Francisco, San Francisco, CA, United States of America; University of New South Wales, AUSTRALIA

## Abstract

**Background:**

Numerous studies have evaluated the efficacy of interventions to reduce risk for sexually transmitted infections in adolescents in recent years, but their global effects remain unknown since 2008, the last date of a comprehensive review of prior studies.

**Aims:**

This study aims at evaluating the efficacy of interventions to promote sexual health, reduce STIs and unplanned pregnancies targeted to adolescents available after 2008; and analyzing the moderators of their global efficacy.

**Methods:**

We searched electronic databases and manual searches of some journals focused on STIs in May 2016. The studies evaluated the efficacy of interventions to reduce sexual risk in adolescents (age range: 11–19) anywhere in the world. Effect size of the relevant outcomes for sexual risk was calculated for every study. Analyses incorporated random-effect assumptions for each outcome. The homogeneity in the results was examined with the *I*^*2*^ statistic and its associated 95% confident interval.

**Results:**

Data from 63 studies (59,795 participants) were analyzed for behavioral and non-behavioral outcomes. In the short term, interventions had a positive impact in sexual health-related knowledge (Hedges’*g* = 1.01), attitudes (*g* = 0.29), self-efficacy toward condom use (*g* = 0.22), intention to refuse sex (*g* = 0.56), condom use intention (*g* = 0.46), and condom use (*g* = 0.38). In the medium term, positive effects observed at the short-term were maintained, although effect size significantly decreased in all variables. In the long term, interventions improved condom use (*g* = 0.47). Moderators of the efficacy are discussed.

**Conclusions:**

Sexual health promotion interventions are effective to promote sexual health through increasing condom use. Effects on non-behavioral variables tend to decrease over time, while condom use increased in the long-term. Interventions should focus on the long-term efficacy, especially in behavioral and biological measures.

## Introduction

Adolescents remain highly vulnerable to sexually transmitted infections (STIs) [1]. Gobally, it is estimated 2.1 million adolescents aged 10–19 are living with HIV in 2012 [2]. A total of 333 million of curable STIs are acquired every year worldwide; adolescents aged 15–19 years old represent the second group with the highest rate of STIs (after the 20–24 year old group) [3]. Excluding HIV and other viral infections, one in 20 young people may contract an STI each year, which increases the likeliness of acquiring HIV [3]. Another problem arising from unprotected sexual behaviors, especially affecting adolescents, is unplanned pregnancies. Every year, 16,000 births from adolescent mothers are registered worldwide [4]. Pregnancy at an early age increases the risk of obstetric problems, such as premature birth and anemia; and it is associated with low psychosocial development of the mother, including attrition from schools and lower labor opportunities [5,6]. Unprotected sex is the main cause of transmission of STIs and unwanted pregnancies. Numerous interventions have been designed to reduce adolescent sexual risk through the promotion of consistent condom use and sexual abstinence.

The efficacy of HIV prevention interventions has been extensively evaluated by meta-analytic and systematic reviews [7–16]. For example, Protogerou and Johnson [17] conducted a meta-review on the factors underlying the success of behavioral HIV-prevention interventions for adolescents. Instead of examining source documents, this study reviewed five meta-analyses and six systematic reviews. The authors concluded that HIV prevention interventions have positive effects on HIV-related knowledge, subjective norms, abstinence, delaying sexual intercourse, decreasing the number of partners, and condom use. Often, prior reviews meta-analytic and systematic reviews have provided evidence for the efficacy of interventions to reduce sexual risk in adolescents for only specific geographic locations, such as low- [18,19] or high-income countries [20], Latin American countries [14], the United States [21], Europe [22], and/or specific populations such as African Americans [13]. Prior reviews have also focused on a specific type of intervention, such as computer technology-based interventions [15,23]; or different characteristics of the intervention, such as the intensity of the program [18]. However, results from these prior reviews cannot be extrapolated to the global adolescent population.

In a global meta-analysis of interventions to reduce sexual risk for HIV in adolescents, Johnson et al. [24] examined 67 studies (98 interventions) available from 1985 to 2008 as well as findings from a previous meta-analyses of studies available from 1985 to 2000 [25]. The authors concluded that these interventions were able to reduce the incidence of STIs through condom use. The results indicated that HIV prevention interventions were successful in increasing: condom use, skills to negotiate protection methods, communication about sex with sexual partners, and to postpone and/or reduce the frequency of sexual intercourse. In addition, these meta-analyses identified important moderators of intervention efficacy [24,25]. Results indicated high variability in the factors that are related to the success of preventive actions over time. Both of these meta-analyses [24,25] were particularly significant in that they were not delimited by geographic area or focus exclusively on risk adolescent population (as adolescents living in depressed areas, drug users, etc.).

The current meta-analysis aims to extend the work of Johnson et al. [24,25] by analyzing the efficacy of interventions to promote sexual health and prevention of STIs, including HIV and pregnancies, in studies available since 2008, the last date of inclusion of the prior studies. Therefore, we will include in our analysis: (1) interventions to promote sexual health, and (2) other relevant outcomes, such as knowledge, attitudes and intention, besides sexual behaviors, skills and STI rates.

This analysis also extends prior research by examining the efficacy of interventions based on sample characteristics (gender, age, HDI, application setting), intervention methodology (based on a theoretical approach or not, promotion of abstinence, parent’s participation), and the evaluation methodology (design of the study, the inclusion of a control group or not, and control group receiving an intervention equivalent to the experimental group but without sexual health contents or non-intervention control group). Following previous meta-analysis of intervention efficacy [26], we analyzed studies based on the short- (posttest assessment), medium- (12–18 month follow up), and long-term effects (24 months or longer follow up) of the interventions. The success of the interventions was determined by the extent to which they increased the number of protected sexual encounters (increased condom use) and objective measures (STI and pregnancy). We also examined other non-behavioral outcomes related to sexual risk that were included in the main theoretical models for promoting health behaviors [27–29], such as sexual health-related knowledge, attitudes towards condom use and safe sex, subjective norms, self-efficacy to use condoms, abstinence self-efficacy, communication about sex with the sexual partner, intention to use condoms during sexual intercourse and to remain sexually abstinent.

## Method

The present study was approved by the Ethics Committee of the Miguel Hernández University (Ref. DPS-JPE-001-10). This research was completed in accordance with the Preferred Reporting Items for Systematic Reviews and Meta-Analyses (PRIMSA; S1 PRISMA Checklist).

### Data sources and searches

Searches for studies were performed using several strategies: (1) Searches in electronic databases (MEDLINE/PubMed, Web of Knowledge, Scopus, PsycINFO, ERIC, Tripdatabase, Google Scholar, Cochrane, and Teseo) through May 2016. (2) Requests were sent to active researchers in the area of sexual-health promotion and HIV prevention with adolescents. (3) Manual searches on some journals focused on STIs and the evaluation of preventive interventions in May 2016 (e.g., *African Journal of AIDS Research*, *AIDS*, *AIDS Research and Treatment*, *AIDS and Behavior*, *AIDS Education and Prevention*, *American Journal of Public Health*, *Evaluation and Program Planning*, *Health Communication*, *Health Education Research*, *Health Policy and Planning*, *Health Psychology*, *Journal of Acquired Immune Deficient Syndromes*, *Journal of Sex Research*, *Journal of the Association of Nurses in AIDS Care*, and *Social Science & Medicine*). Studies that met the selection criteria and were available by May 31^st^ 2016 were included. The following retrieval indexes were used: ((adolescent* OR teen* OR young*) AND (sexually transmitted diseases OR STD OR sexually transmitted infections OR STI OR pregnan* OR unplan* pregnan* OR AIDS OR HIV OR sexual* risk* OR sexual* behavior*) AND (condom use OR method of protection OR attitude OR knowledge OR intention OR self-efficacy OR promot* OR prevent*) AND (effectiv* OR efficac* OR evalua*) AND (educat* OR primary prevention OR randomized controlled trial OR RCT OR controlled clinical trial OR CCT OR intervent* OR program* OR control trial OR school-based OR meta-analysis OR systematic review). In addition, reference lists of the included articles were cross-checked to search for additional relevant studies that were not detected by the original literature search.

### Study selection

Studies had to meet the following inclusion criteria: (1) evaluate interventions aimed at reducing HIV, STIs, unplanned pregnancies, or promote a healthy sexuality, (2) target adolescents aged 11–19, (3) include relevant STI risk behavior variables or precursors of sexual risk (e.g., knowledge, attitudes, etc.), (4) offer outcomes in terms of pre-post change or a between-groups post-intervention comparison, (5) provide sufficient statistics to calculate effect sizes (ES), and (6) be published or available from 2008 to 2016.

**Fig 1:**
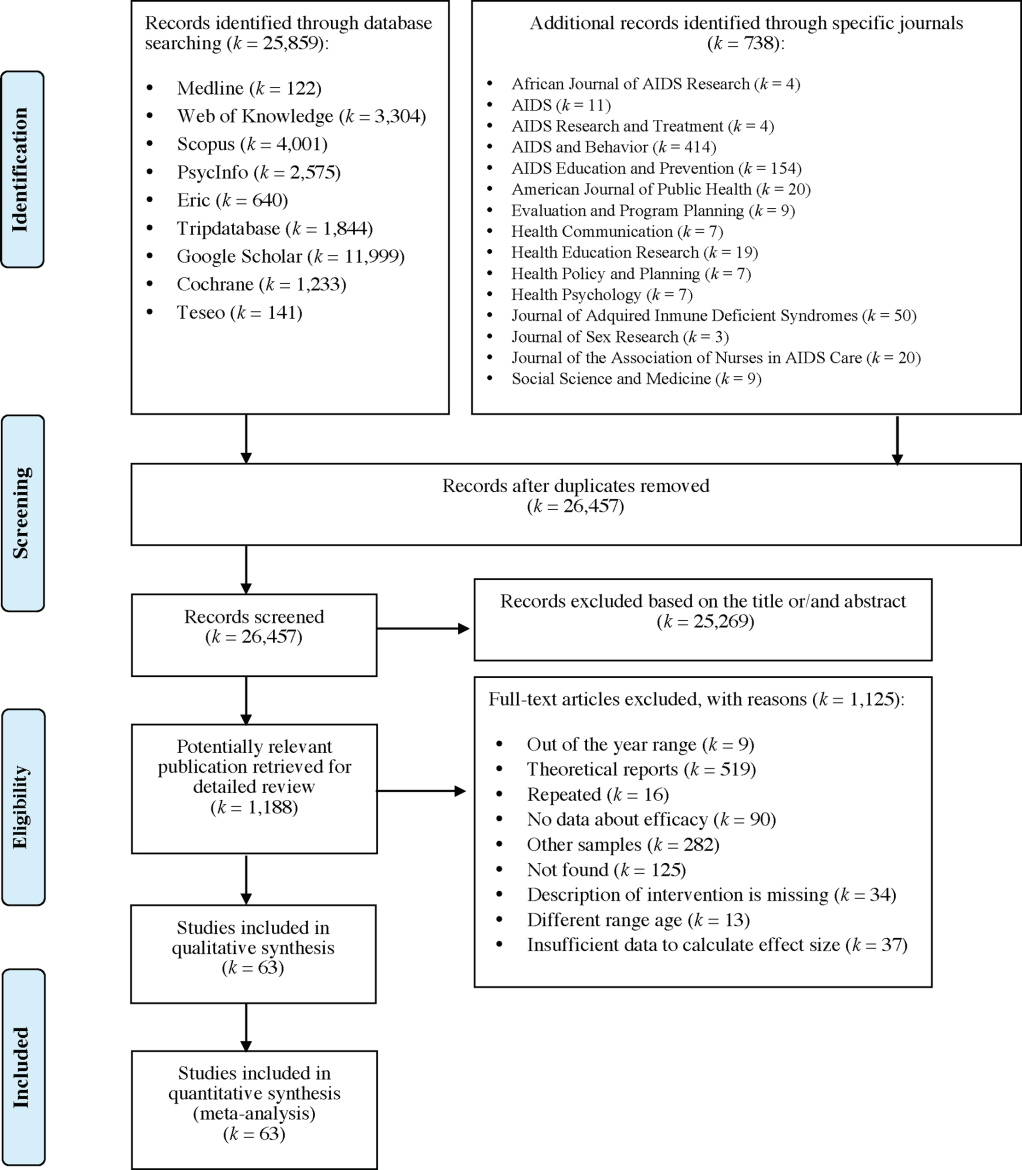
Flowchart of the report selection process.

Following Fonner and colleagues [19], no exclusion criteria related to the experimental design (randomized controlled trials or RCTs, non-randomized, before-after) were applied, in order to include as many studies as possible. Excluded were studies reporting: (1) samples aged 20 years or older, or studies that did not disaggregate results for participants aged 11 to 19; (2) samples diagnosed with mental health disorders and/or physical illness (e.g., depression); or (3) samples selected because they were at high-risk for contracting HIV, STIs, and unplanned pregnancies (e.g., drug users or adolescents from marginal settings). From the literature, 26,457 studies were identified of which 63 studied were included, using a sample of 59,973 adolescents aged 11–19 (Fig 1). Only two of the 20 experts invited to send their papers responded and neither of these two studies provided met the inclusion criteria.

### Study information

Screening and coding of studies was done manually. During the screening process, two reviewers independently excluded 25,269 records based on the title or abstract. There were 1,188 studies, of which most were discarded for not meeting the criteria (Fig 1). Initially, 44 studies were discarded because they did not provide sufficient statistics to calculate ES. We contacted the principal author of studies that provided insufficient statistics to calculate ES; only 7 provided us the needed information (*k* = 37 studies were discarded for this reason). Data from 63 studies were extracted by two independent coders and introduced in the database following the study protocol. The Human Development Index (HDI) for each study was coded according to country and year of publication. This social indicator ^_^ developed by the United Nations Development Programme (UNDP) ^_^ consists of three parameters: lifelong healthy (life expectancy at birth), education (mean and expected years of schooling), and standard of living (gross national income [GNI] per capita) [30]. The index ranges between 0 and 1, with higher scores indicating greater degree of human development. Following Huedo-Medina et al.’s [14] procedure, when the year of publication was not specified, the next year’s with HDI data available was taken. To calculate the reliability, all studies were screened and coded by another encoder independently. Spearman-Brown formula was used to calculate the reliability for continuous variables, and *Kappa* (*κ*) for categorical variables. Reliability was good, ranging from .90 to 1, with a mean of .96 across categories (Table 1). Discrepancies were resolved by a third reviewer, following the procedure described by Picot et al. [31].

**Table 1:** Descriptive features of 63 studies in the sample.

Feature (reliability)	Values (%)
Year of publication (*r* = .95)	
Mean	2012
Median	2012
*SD*	2.53
Country (κ = .92)	
United States	31 (49.2)
Spain	5 (7.9)
South Africa	5 (7.9)
China	3 (4.8)
Cuba	3 (4.8)
United Kingdom	2 (3.2)
Mexico	1 (1.6)
Uganda	1 (1.6)
Trinidad and Tobago	1 (1.6)
Thailand	1 (1.6)
Liberia	1 (1.6)
Nigeria	1 (1.6)
Panamá	1 (1.6)
India	1 (1.6)
Colombia	1 (1.6)
Canada	1 (1.6)
Bahamas	1 (1.6)
South Korea	1 (1.6)
Puerto Rico	1 (1.6)
Human Development Index	
Very high	40 (63.5)
High	12 (19)
Medium	6 (9.5)
Low	5 (7.9)
Intervention time (*κ* = 1)	
Less than 4h	7 (14.6)
4h or more	41 (85.4)
Design	
Randomized control trials	25 (39.7)
Cluster-randomized control trial	20 (31.7)
Non-randomized control trial	18 (28.6)
Intervention based on theories (κ = .90)	45 (71.4)
Methodology (κ = 1)	
Passive	4 (7.3)
Interactive	51 (92.7)
Assessments (in months)	
Short-term	
Mean (*SD*)	1.03 (1.81)
Range	0–6
Medium-term	
Mean (*SD*)	12.70 (1.99)
Range	12–18
Long-term	
Mean (*SD*)	29.50 (7.54)
Range	24–40
Goals of the intervention	
HIV	54 (88.5)
STIs	36 (72)
Pregnancy	34 (79.1)
Sexual health promotion	43 (84.3)
Intervention included	
HIV prevention	54 (94.7)
STIs prevention	35 (77.8)
Pregnancy prevention	32 (80)
Male condom	41 (87.2)
Female condom	3 (10)
Sexual abstinence	28 (44.4)
Transmission routes	31 (83.8)
STIs effects	21 (65.6)
People living with VIH	13 (52)
Drugs prevention	8 (26.7)
Social skills training	16 (53.3)
Auto-instructions	10 (35.7)
Emotional management	10 (33.3)
Self-esteem	9 (29)
Parents’ participation (κ = 1)	
No	54 (85.7)
Yes	9 (14.3)
School as setting of implementation	55 (94.8)
Participant characteristics (*k* = 63)	
*N* at posttest (*r* = .94)	
Total	38,880
Mean	1087.69
Median	784
*SD*	1314.41
% females (*r* = .98)	58.26
% sexually active	39.37
Average age (*r* = 1)	
Mean	14.96
Median	15.01
*SD*	1.37

*k* = number of studies; *SD* = Standard Deviation. Methodology: 1) Passive: When adolescents simply receive instruction through explanatory talks or watching videos, without their participation being pursued; 2) Interactive: Includes strategies to promote the participation of the members of the group (e.g. sharing opinions, dynamic groups, social skills training through role-playing). Emotional management: Refers to the inclusion of strategies for the emotional management (e.g. relaxation, breathing exercises to relax, etc.).

In order to determine the effect of the interventions, the ES–Hedges' *g* of the relevant outcomes for sexual risk–was calculated for every study. Consistent with Fonner et al. [19], we calculated Hedges' *g* coefficients using Comprehensive Meta-Analysis (CMA) and following the procedures developed by Hasselblad and Hedges [32]. The interpretation of Hedges' *g* is similar to Cohen’s *d*, whereby effect sizes can be interpreted as small (*d* = 0.20), medium (*d* = 0.50), and large (*d* = 0.80) [33,34]. The outcomes included were: knowledge, attitudes, subjective norms, self-efficacy to use condoms, abstinence self-efficacy, communication about sex with the sexual partner, condom use intention, intention to refuse sex, condom use, and pregnancy and STIs rates; the sign of *g* was set so that reductions in risk were positive (i.e., improvements). We performed relevant transformations when means and standard deviations were not provided. Following Johnson et al. [24], data from the main intervention and control group were used in the studies evaluating the efficacy of two or more interventions compared to a control group. The interventions that included novel contents (including a novelty from a previous validated version) were considered as the main intervention. For example, in the Morales et al. [35] study, the intervention including peers as co-facilitators was considered the main intervention (compared to the traditional version of the intervention). The effect of short-term interventions (posttest), medium-, and long-term were evaluated separately. Every follow-up was codified separately in the database. For intragroup studies, pre- and post/follow-up measures were used to calculate ES; while for controlled studies, measures of the intervention and control groups were used.

Analyses incorporated random-effect assumptions for each outcome, using restricted maximum-likelihood estimators in the *metafor* package for *R* [36]. The homogeneity in the results was examined with *I*^*2*^ statistic (percent) and its associated 95% confidence interval (*CI*). Conventionally, high heterogeneity corresponds to percentages of around 75%, medium with values around 50%, and low with values around 25% [37]. Egger’s regression test was used to examine the possibility of publication bias. Egger’s test estimates the extent in which asymmetry is present in a distribution of effect sizes; and it is also one of the most popular quantitative methods to examine asymmetries [38,39]. The average quality of the studies was calculated according to eight criteria, based on previous studies [26,40]: type of design, randomization, attrition at posttest, attrition at the follow-up, evaluator blind procedure, use of validated measures for targeted population, equivalence of a control group, sample sizes. Descriptive analyses were conducted using SPSS v23.

## Results

### Descriptive outcomes

The analyses included 63 studies published between 2008 and 2016 [35, 41–102] (Fig 1 and S1 Table). Half of the studies in the sample were conducted in the United States. Five studies were conducted both in Spain (7.9%) and in South Africa (7.9%). Three studies were respectively from China (4.8%), and Cuba (4.8%). Two studies were conducted in United Kingdom (3.2%). The rest of studies were conducted in other countries (Table 1). The majority of the studies were conducted in either North or South America (66.1%); the 53.2% represents North America, including Canada and Trinidad and Tobago. Africa was the setting for the 12.9% of the studies, 11.3% were in Europe and 9.7% were in Asia. As seen in Table 1, the mean HDI for countries in the sample during the year of publication was .82 (*SD* = .13), with a range from .41 to .92. The mean of the very high HDI countries ranged from .83 to .91 (*Mean* = .90, *SD* = .01), with the highest indexes for United States, Canada, and United Kingdom. The mean of the high HDI countries such as Panamá and South Korea ranged from .70 to .79 (*Mean* = .74, *SD* = .03). The mean of the medium HDI countries such as India and South Africa ranged from .60 to .66 (*Mean* = .65, *SD* = .02). Low HDI countries included Liberia (.41), and Haiti (.47), Uganda (.48), and Nigeria (.49); the *Mean* was .46 (*SD* = .03).

The sample comprised 59,795 adolescents at pretest, with 23,618 in control conditions and 36,177 in experimental conditions, with a mean age of 14.96 (*SD* = 1.37). When the study did not include control group, participants were considered as part of the experimental group receiving the intervention. About 40% of the participants were sexually experienced. In most cases, the intervention was carried out at school (*N* = 55). Few studies combined different settings of implementation, such as García et al. [64], who conducted their study in Cuba and recruited students in the School of Medical Science (*N* = 358); some activities were conducted at the parks, streets, hospitals or other places where participants could learn about sexual health promotion. Interventions tended to focus on HIV, STIs, pregnancy prevention, and/or sexual health promotion. The duration of the interventions ranged from 1 to 68 weeks (*Mean* = 10.78, *SD* = 12.36). More than half of the interventions (73%) were based on theories of health promotion. Social cognitive therapy, the theory of reasoned action, and the theory of planned action were the most used theoretical models, which is consistent with the meta-analysis conducted by Robin et al. [16]. Only 12.7% (*k* = 8) of the studies included the adolescents’ parents in the intervention.

Of the 63 analyzed studies, 25 (39.6%) reported to assign randomly the participants to the experimental conditions (RCTs), which is considered high-standard assignment in research because it minimizes selection bias. There were 20 cluster-randomized control trials (31.7%), and in most of the cases, schools were assigned to the experimental condition. Only 28.6% of the studies did not use randomization as assignment method. Of the 45 studies including a control group, 30 (66.6%) controlled for Hawthorne effects by implementing an alternative intervention to the control groups. Control groups were usually health promotion interventions (basically drug avoidance, diet, exercise, and family life education) [46,47,49,75,93,100]. In some cases, adolescents assigned to the control group received sexual health and/or HIV prevention content [45,72,95,101], but the intervention did not include active health promotion activities. For example, Armitage and Talibudeen [45] provided information on the history of the condom to the participants assigned to control group. Jones et al. [72] used a passive methodology for the comparison group, which consisted of watching three 25–30 min-DVDs about HIV/AIDS (*Force Ripe Man Part1-2*, *Understanding HIV/AIDS*, and *Voices*). Treatment as usual or the traditional sexual health promotion intervention offered by the school was implemented as the control condition in a few studies [45,72,95]. See Table 1 for more information on the main descriptive characteristics of the included studies. The average quality of the studies was acceptable (*Mean* = 3.17, *SD* = 2.02; range: 0 to 6.50 out of 8 possible). There were no statistically significant differences in the quality of the studies across the countries of implementation (*p* = .80) nor HDI (*p* = .22).

### What was the effect of the interventions on the outcomes?

#### Short-term outcomes

**Fig 2:**
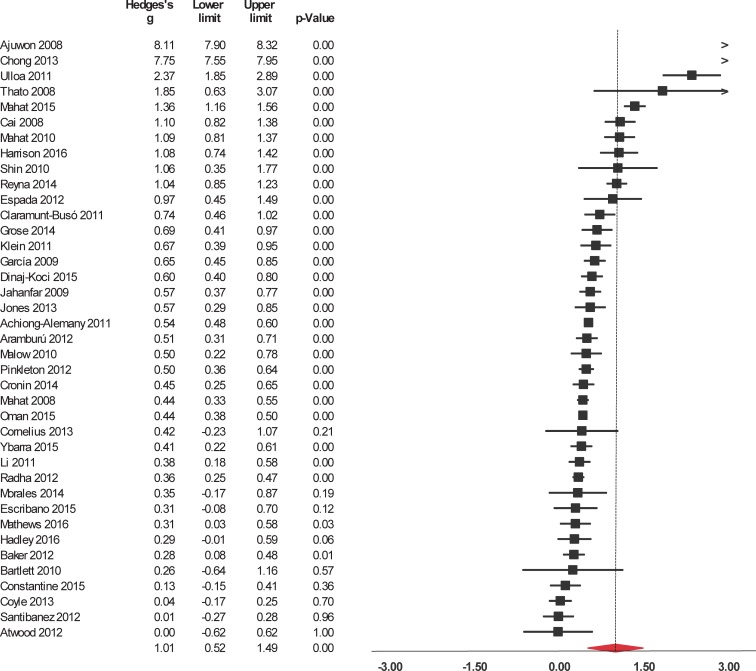
Forest plot from meta-analysis of HIV-related knowledge (short-time). Effects are ordered from most successful to least successful. The overall estimate for each is represented at the end of the list of studies.

**Fig 3:**
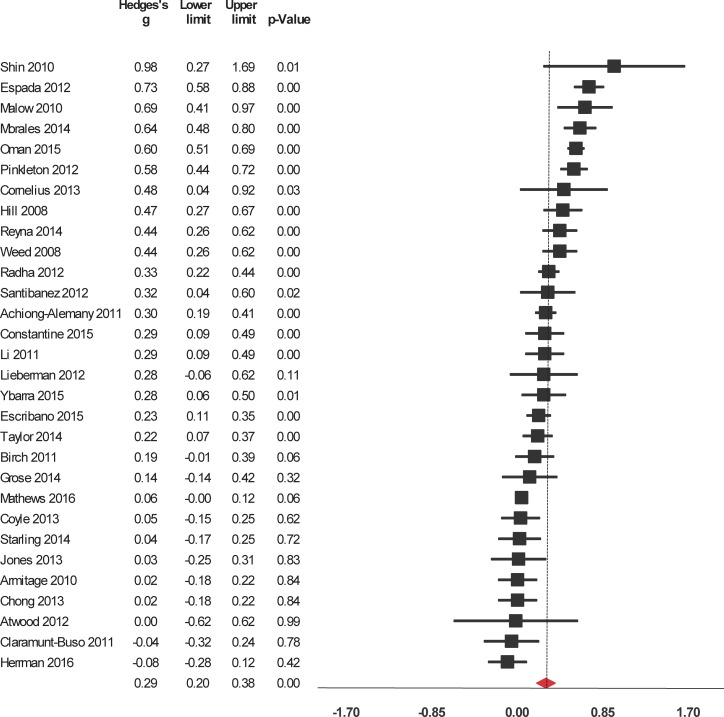
Forest plot from meta-analysis of attitudes (short-time). Effects are ordered from most successful to least successful. The overall estimate for each is represented at the end of the list of studies.

**Fig 4:**
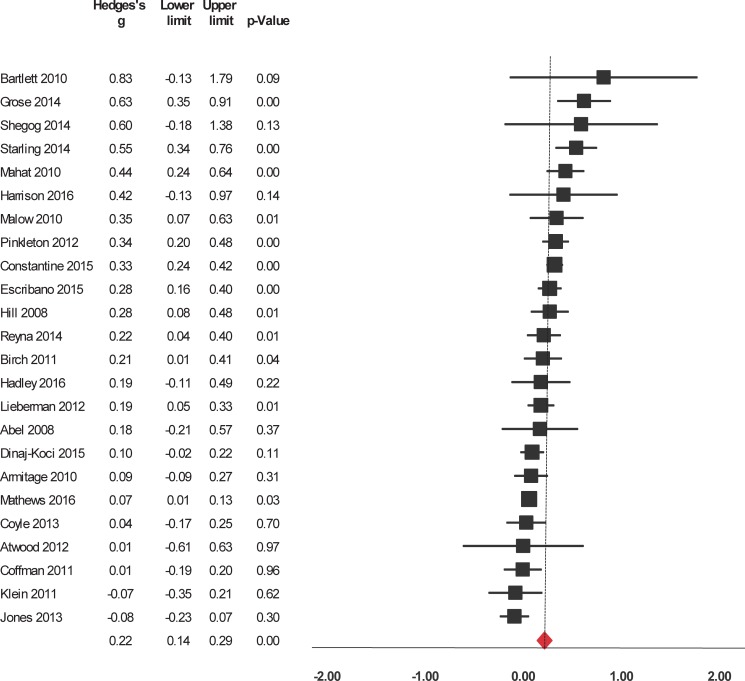
Forest plot from meta-analysis of self-efficacy to use condoms (short-time). Effects are ordered from most successful to least successful. The overall estimate for each is represented at the end of the list of studies.

**Fig 5:**
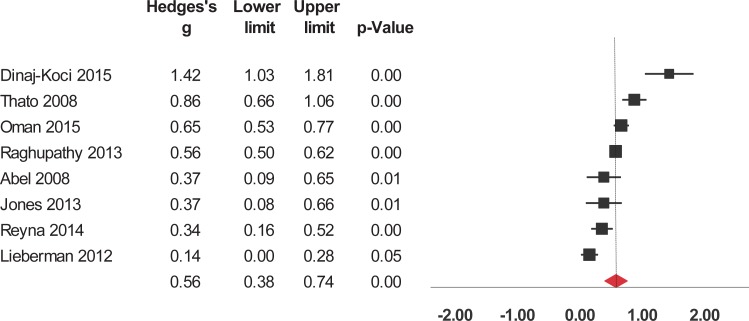
Forest plot from meta-analysis of intention to refuse sex (short-time). Effects are ordered from most successful to least successful. The overall estimate for each is represented at the end of the list of studies.

**Fig 6:**
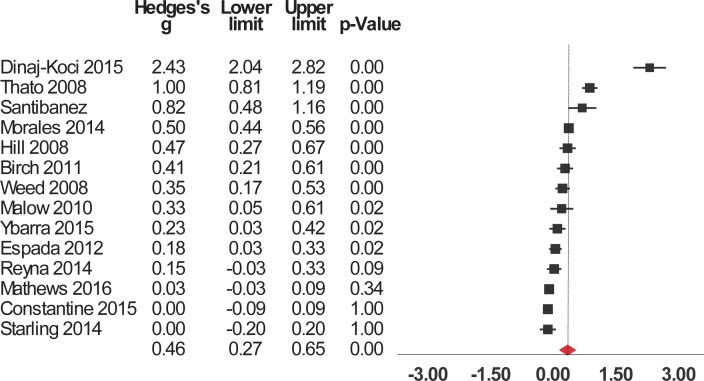
Forest plot from meta-analysis of condom use intention (short-time). Effects are ordered from most successful to least successful. The overall estimate for each is represented at the end of the list of studies.

**Fig 7:**
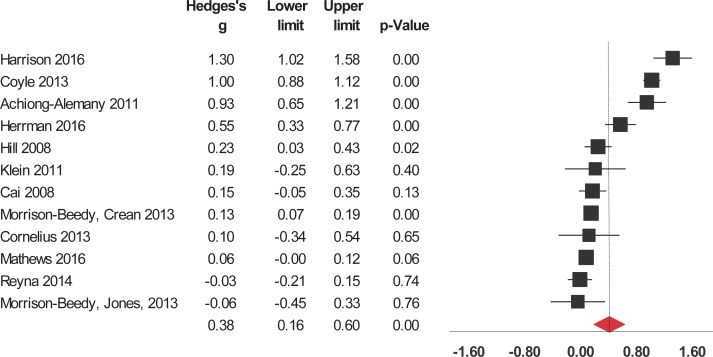
Forest plot from meta-analysis of condom use (short-time). Effects are ordered from most successful to least successful. The overall estimate for each is represented at the end of the list of studies.

Interventions significantly enhanced 6 of the 9 evaluated outcomes (Table 2). The studies were widely heterogeneous in the outcomes selected. For short-term outcomes, interventions significantly increased knowledge about HIV/AIDS and its routes of transmission, condom use, contraceptives, other STIs (but not including HIV), and sexual health in general (Fig 2). The interventions also had a significant, positive short-term impact on attitude towards sexual health including HIV, preventing pregnancy, and beliefs about abstinence, condom use, and people who have sex with same sex (Fig 3). Interventions also increased and self-efficacy to use condoms (Fig 4) and behavioral intentions, including intention to refuse sex (Fig 5) and intention to use condoms (Fig 6). No impact on subjective norms, abstinence self-efficacy, and communication about sex with the sexual partner was found in the short-term. Interventions increased condom use (Fig 7). Only one study indicated a reduction in self-reported STIs among adolescents that were already sexually active at baseline; however, the intervention had no impact on pregnancy rate [53]. It is important to consider how the high heterogeneity across the studies may influence interpretation of results, and moderators are described below.

**Table 2:** Weighted *Mean* effect sizes and related statistics at short-, medium- and long-term assessments for interventions targeting adolescents.

		Hedges’ *g*		*I*^2^
Outcome	*k*	95% *CI*^a^	*p*	(95% *CI*)^b^
Knowledge				
Short-term	39	1.01 (0.52 to 1.49)	.0002	99.69 (99.54 to 99.81)
Medium-term	12	0.40 (0.26 to 0.54)	< .0001	88.65 (75.83 to 95.52)
Long-term	3	0.19 (-0.07 to 0.46)	.14	64.09 (0 to 98.87)
Attitudes				
Short-term	30	0.29 (0.20 to 0.38)	< .0001	90.53 (83.78 to 95.30)
Medium-term	7	0.08 (0.02 to 0.14)	.007	24.19 (0 to 68.94)
Long-term	3	0.09 (-0.09 to 0.28)	.31	0 (0 to 0)
Subjective norms				
Short-term	13	0.06 (-0.01 to 0.14)	.11	65.69 (30.66 to 91.33)
Medium-term	6	0.33 (-0.26 to 0.93)	.27	98.88 (96.83 to 99.86)
Long-term	2	0.01 (-0.16 to 0.19)	.88	0 (0 to 99.35)
Self-efficacy to use condoms				
Short-term	24	0.22 (0.14 to 0.29)	< .0001	75.69 (54.46 to 89.54)
Medium-term	9	0.08 (0.007 to 0.15)	.03	52.01 (0 to 80.53)
Long-term	3	0.17 (-0.03 to 0.35)	.051	0 (0 to 27.40)
Abstinence self-efficacy				
Short-term	4	0.10 (-0.06 to 0.27)	.22	70.35 (8.64 to 97.23)
Medium-term	4	0.05 (-0.09 to 0.20)	.47	83.45 (40.22 to 98.94)
Long-term	2	0.09 (-0.39 to 0.59)	.69	0 (0 to 14.43)
Communication about sex with the sexual partner				
Short-term	4	0.32 (-0.19 to 0.83)	.21	95.31 (84.30 to 99.67)
Medium-term	2	0.04 (-0.12 to 0.22)	.58	0 (0 to 99.66)
Long-term	0			
Intention to refuse sex				
Short-term	8	0.56 (0.38 to 0.74)	< .0001	95.23 (88.23 to 99.06)
Medium-term	2	0.04 (-0.04 to 0.13)	.31	0 (0 to 89.19)
Long-term	1	0.14 (-2.20 to 2.48)	.90	-
Condom use intention				
Short-term	14	0.46 (0.27 to 0.65)	.002	98.70 (97.51 to 99.54)
Medium-term	7	0.08 (0.02 to 0.14)	.003	0 (0 to 67.13)
Long-term	0	—	—	—
Condom use				
Short-term	12	0.38 (0.16 to 0.60)	.002	97.35 (94.37 to 99.10)
Medium-term	9	0.29 (0.06 to 0.53)	.004	96.39 (89.91 to 98.96)
Long-term	2	0.47 (0.28 to 0.66)	< .0001	83.37 (16.53 to 99.98)

Follow up 1 = includes studies that evaluate the efficacy of the intervention between 12 and 18 months. Follow up 2 = includes studies that evaluate the efficacy of the intervention at 24 months and after. *k* = number of studies included in the analyses. *I*^2^ = consistency of effect sizes.

ª Estimates of effect size values are greater than 0 (*d*) for differences in favor of reduced risk for the intervention group.

^b^ Values range from 0 (homogeneity) to 100 (heterogeneity), assessed using random-effects assumptions.

#### Medium-term outcomes

**Fig 8:**
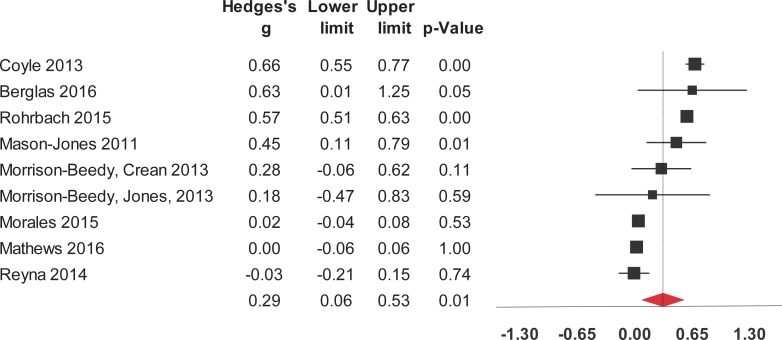
Forest plot from meta-analysis of condom use (medium-time). Effects are ordered from most successful to least successful. The overall estimate for each is represented at the end of the list of studies.

Interventions to reduce risk for STIs in adolescents had a significant impact on 5 of the 9 outcomes evaluated in the studies’ medium-term measures (Table 2). These interventions significantly and positively impacted on knowledge about HIV/AIDS and its routes of transmission, condom use, contraceptives, other STIs (no-HIV). The interventions also had a significant and positive impact on sexual health in general and attitude towards sexual health including HIV, preventing pregnancy, and beliefs about abstinence, condom use, and people who have sex with same sex. Positive effects were found for self-efficacy to use condoms, condom use intention, and condom use (Fig 8). In the medium-term, interventions had no significant impact on variables on the rest of studied outcomes. There were too few studies that provided medium-term data in order to run detailed models.

#### Long-term outcomes

Interventions to reduce risk for STIs in adolescents had a significant impact only in condom use in the studies’ long-term measures (Table 2). Interventions did not have impact on variables on the rest of studied outcomes. Only two studies reported long-term measures for condom use, which prevented fitting detailed models.

### Publication bias

In examining condom use outcomes, Egger’s regression test was not significant at posttest (*z* = 1.17, *p* = .23), which suggests no evidence of publication bias. However, evidence suggestive of publication bias was found in the medium-term (*z* = 11.57, *p* ≤ .001) and long-term (*z* = -3.23, *p* = .001). An important caveat is that there is heterogeneity in the effect sizes, and that therefore, other factors are likely influencing their magnitude.

### What characteristics of the intervention and sample explain variations in condom use outcomes in the medium-term?

Because of the scarcity of study that evaluates condom use at long-term (*k* = 2), moderator analyses was conducted using medium-term data related to condom use, the longest follow-up available. All studies that evaluated condom use in the medium term (12–18 months) included a control or comparison group; therefore, control group was not included as moderator in the analyses. As Table 3 shows, seven of the 10 moderator dimensions evaluated were statistically significant moderators of condom use. Relative to the characteristics of the intervention and sample, the interventions to reduce risk for STIs and pregnancy in adolescents were more effective in increasing condom use when: 1) the study took place in a nation with a very high HDI (vs. low HDI), 2) the study was implemented in school settings (vs. other settings), 3) interventions were based on a theoretical approach, and 4) interventions did not promote sexual abstinence. Gender and age of the participants were not moderators of the efficacy of the interventions. Interventions appeared to increase rates of condom use when they involved parents (Hedges *g* = 0.63; 95% CI: 0.01, 1.24), but the test of moderation was not statistically significant (*p* = .12).

**Table 3:** Estimates of condom use effect sizes as a function of moderator dimensions and showing sub-groups for moderators that are statistically significant.

Dimension and level	*Q*_*Model*_	*p*	Hedges’ *g* (95% *CI*)
**Sample characteristics**			
Gender (*k* = 9)	0.49	.48	-0.004 (-0.01 to 0.007)
Age (*k* = 8)	0.004	.94	-0.007 (-0.23 to 0.21)
Human development index (HDI) (*k* = 9)	8.43	.01	
Very high			0.33 (0.09 to 0.57)*
High			—
Medium			—
Low			0.18 (-0.22 to 0.60)
Setting of intervention (*k* = 9)	7.37	.02	
School			0.29 (0.07 to 0.52)*
Other			0.28 (-0.37 to 0.93)
**Intervention methodology**			
Based on theoretical model (*k* = 9)	8.06	.01	
Yes			0.28 (0.06 to 0.50)*
No			0.45 (-0.20 to 1.10)
Promotes abstinence (*k* = 9)	7.97	.01	
Yes			0.21 (-0.14 to 0.57)
No			0.34 (0.08 to 0.59)**
Parental participation (*k* = 3)	4.08	.12	
Yes			0.63 (0.01 to 1.24)*
No			0.04 (-0.21 to 0.29)
**Evaluation methodology**			
Design of the study (*k* = 9)	8.06	.01	
Randomized control trial			0.28 (0.06 to 0.50)*
Cluster-randomized control trial			—
Non-randomized			0.45 (-0.20 to 1.10)
Control receives a weakened dose intervention (*k* = 7)	11.08	.004	
Yes			0.008 (-0.35 to 0.37)
No			0.41 (0.17 to 0.65)*
Year of publication (*k* = 9)	0.65	.41	-0.05 (-0.18 to 0.07)

**Note.** Each moderator is evaluated on a bivariate basis, without controlling for the other features. *CI* = confidence interval

*p ≤ .05

**p ≤ .01.

Regarding the evaluation methodology, the interventions increased condom use more when: 1) the study randomly assigned participants into an experimental group or a control group (RCT) vs. using non-random assignment, and 2) the control group did not receive an alternative intervention. The impact of the interventions on adolescents’ condom use did not increase over time, which means that the year in which the study was published was not a moderator of the efficacy of the intervention to increase condom use in the medium term.

## Discussion

The present meta-analysis summarized the efficacy of interventions for STI prevention and sexual health promotion for adolescents performed in recent years (2008–2016), and identified the moderators of the efficacy of such interventions to increase medium-term condom use. The results indicated that interventions had the biggest and more reaching impact in the short-term. The interventions were effective in increasing sexual health-related knowledge, promoting a favorable attitude towards HIV and methods of protection, self-efficacy to use condoms, behavioral intention including condom use intention and intention to refuse sex, and increasing condom use among adolescents. Intervention effect size magnitudes were low to moderate, except for knowledge, which was high [33]. These results are consistent with previous meta-analyses [17,24,31,103] and, as with these same meta-analyses, overall heterogeneity was large (Table 2). Our team was successful at applying several a priori moderators in models of the medium-term condom use effect size (Table 3), and we comment on these results below.

The lack of impact of interventions on subjective norms (Table 2) may be related to the fact that participants’ close friends usually do not attend the intervention, so it would not be expected that participants’ close friends increase their condom use after the intervention; therefore, the participants’ perception of peers’ condom use tend to be stable over time, as suggested by Jemmott et al. [104]. This unexpected result suggests that the subjective norms component needs to be greater attention as it is one of the precursors of condom use according to multiple theories [29,105] and empirical studies [61]. For example, the Network-Individual-Resource (NIR) model for HIV prevention highlights how exchanges of resources between individuals and their networks underlies and sustains HIV-risk behaviors [106]. Exceptionally, the COMPAS intervention–implemented in Spanish schools–had an impact on subjective norms one year after its implementation [83].

The short-term effects of the interventions paralleled those that were significant at the medium term (12–18 months’ post-intervention), except for intention to refuse sex and communication about sex with the sexual partner. However, the interventions’ effect in every outcome decreased over time, which suggests that short-term effects observed in variables related to sexual risk tend to decline over time. This pattern remained in the long-term effects (24 months’ post-intervention and after), where interventions were successful only at increasing condom use. Only two studies provided long-term condom use effects after the 24-month follow up [80,101]. Because of the lack of monitoring data for the medium- and long-term, we cannot draw firm conclusions. These patterns are consistent with the systematic review of school-based cognitive-behavioral interventions conducted by Kavanagh et al. [107].

Interventions tended to succeed better when they took place in nations with higher HDI, such as United States, Canada or Spain. This finding conflicts with results from a meta-analysis of 37 interventions for HIV prevention (28 studies) applied in Latin American and Caribbean Nations (from 1995 to 2008); specifically, Huedo-Medina et al. [14] found greater efficacy to increase condom use in interventions implemented in countries with low or medium HDI. According to these authors [14], the conditions of poverty and deprivation imply resource deficits that the interventions address, in part. Yet this review did not target studies of adolescents, and it is true that in all societies, adolescents are sheltered by parents and to some extent schools and peer groups, which can be supportive to prevent sexual risk behaviors. These networks tend to be stronger in high HDI nations, which may explain our results. Another variable that may explain the relationship between HDI and intervention efficacy is community-level stigma. Reid, Dovidio, Ballester and Johnson [108] found that in communities with high levels of stigma, HIV prevention interventions were not successful in increasing condom use. Since stigma has been directly associated with lower socioeconomic and lower educational levels [108], it could be expected that interventions have a lower impact on condom use over time in low HDI nations.

According to other meta-analyses and reviews assessing the efficacy of interventions in adolescents [17,24,31], most studies were conducted in the school context (95%), and had a higher impact in condom use than those delivered in other settings such as streets, health centers, etc. We believe that the availability of schools, the learning context, and the ease access to adolescents, even some time after applying the intervention (monitoring) are factors that make schools a recommendable setting to promote sexual health during adolescence.

The most successful interventions to increase condom use were based in theoretical approaches and did not promote sexual abstinence. Similarly, Johnson et al. [24] concluded that interventions exclusively focused on abstinence were not as effective in reducing sexual frequency as comprehensive interventions including condom use promotion (*d* = 0.10 *vs*. *d* = 0.25). Interventions based on theoretical approaches–such as Social Cognitive Theory and Theory of Planned Behavior–were the most successful at increasing condom use, consistently with Albarracín et al. [9].

Interventions showed greater efficacy when participants were randomly assigned to the experimental conditions (RCT) (compared non-randomized control trials), and when the control group did not received another intervention compared to those receiving an alternative intervention. Findings may be explained by methodological quality of studies. All studies that evaluated condom use in the medium term (12–18 months) included a control or comparison group, which suggests that only controlled studies evaluated interventions’ effects over time. Most of the pre-posttest (intragroup comparisons) studies evaluated the efficacy on condom use at the short term, and this difference may explain why effect sizes for condom use at the short term were generally larger than in the medium term. For example, in a pre-posttest study testing the efficacy of an educational intervention in HIV / AIDS in a sample of 420 Cuban adolescents aged 15–16 [42], the percentage of sexually active adolescents using condom use during penetrative sex increased from 51.4% (pretest) to 94.6% (2-month post-intervention) (*p* < .05). Effects of the interventions that are evaluated without a control group may be overestimated. Pre-post differences can reflect many factors—history, maturation, testing, instrumentation, and so on—other than the intervention itself [109]. All of these factors are less plausible in RCTs, so the results obtained from these designs are trusted more for their greater ability to gauge causal effects. Effect sizes on medium-term condom use were higher when the control group did not receive an alternative intervention. Hawthorne effects–understood as positive changes associated with the attention that adolescents in the experimental group received from researchers–may have inflated effect size in trials [110]. When an additional intervention is applied to the control group, the effects are more likely attributable to the intervention and not to other factors, due to adolescents' awareness of participation [109]. Last, findings showed that the year of publication of the study was not a moderator of the efficacy of the interventions to promote condom use over time, which suggests that this aim (condom use promotion) is not necessary better addressed in the sexual-health promotion interventions nowadays.

### Limitations

This study has some limitations. Of course, the sample was limited to the years 2008–2016, so the results reflect only the most recent studies. The search strategy may have excluded potential papers, although several methods were used to minimize this possibility. Most of the interventions were assessed with self-reports, whose answers may be exposed to recall bias and social desirability. Results must be interpreted taking into account publication bias in the medium- and long-term follow-up analysis. Due to the scarcity of studies evaluating condom use over longer intervals, it was not possible to analyze moderators of the efficacy of the intervention to promote condom use at the 24 months-post intervention and later. In general, the results may have been influenced by the lack of data to calculate ES, poverty in the description of the interventions, samples, and methodology of the evaluation. This limitation is shared by most meta-analysis of efficacy of interventions to promote sexual health and HIV prevention, according to Johnson, Michie, and Snyder’s [12] recent meta-review of meta-analyses. Authors should be aware of the importance of providing relevant information in their reports in order to permit replication of studies and analyses of the overall efficacy of preventive actions. Given this limitation, Mullen et al. [21] proposed a guide elaborated by the American Psychological Association about the basic information regarding the intervention and methodology of the evaluation that authors should provide in their studies.

Finally, In addition, the trials’ measures make it difficult to estimate the effects of interventions on condom use. For example, many studies use a general measure of sexual behavior index to assess the impact of the intervention on different outcomes of sexual behavior, including condom use [53]. The behavioral index involves condom use as well as other sexual behaviors, which makes it difficult to know the specific intervention’s effect on condom use independently of other behavioral outcomes. This limitation has been previously observed in a decade of revision focused on the efficacy of sexual risk reduction interventions [16]. Globally, it is important to note that only 39.37% of participants in our sample of studies were sexually active, which may explain why the researchers selected fundamentally non-behavioral variables in order to assess the efficacy of the interventions. However, level of sexually activity was not a moderator of the efficacy of the interventions.

### Conclusions

In summary, this meta-analysis provides updated knowledge about the efficacy of the interventions that focus on promoting sexual health and preventing HIV infections in adolescents, and the factors that contribute to a greater efficacy of them. These findings are relevant to reduce the economic and human cost on interventions that are not effective. All interventions showed positive effects on all outcomes assessed, although there was a large heterogeneity in the effects. Most of them had a significant impact in non-behavioral and behavioral outcomes in the short-time, but effects tended to decrease over time. The impact in condom use tended to increase over time (at the 24-month follow up and later), which confirms the importance of monitoring behavioral outcomes. Due to the small number of studies that track the effects, firm conclusions about the medium- and long-term efficacy of HIV and sexual-health interventions cannot be drawn. Scarcity of human and material resources for sexual health promotion aimed at adolescents in many countries may be one of the reasons for the lack of studies that include long-term follow-ups and objective measures in their evaluation. As a result, it is difficult to estimate the real benefit of interventions being implemented in the real setting for adolescent sexual health. More funding is needed to adapt interventions that have proven to be effective for high-risk groups. Long-term evaluations are the key to determine whether these interventions actually impact on STIs and unplanned pregnancies during adolescence, and consequently they are reducing associated health and social costs. Future studies should explore long-term effects, especially in behavioral and biological measures. More evidence is needed of the efficacy of sexual health promotion interventions in adolescents recruited from other settings rather than schools, as foster care centers, health centers, and other organizations. Other potential moderators that may be considered in future research are program duration and intensity, program facilitator characteristics, and participant characteristics.

## Supporting information

S1 PRISMA Checklist Preferred reporting items for systematic reviews and meta-analyses (PRISMA) checklist. (DOC)

S1 Table Characteristics of studies included in the meta-analysis (*k* = 63). (DOCX)
