# Supplementary material for: Interventions to reduce risk for sexually transmitted infections in adolescents: A meta-analysis of trials, 2008-2016
Source: PLoS One. 2018 Jun 28;13(6):e0199421. doi: 10.1371/journal.pone.0199421 (PMC6023153; doi:10.1371/journal.pone.0199421)
Supplement: S1 Table — (DOCX) [file pone.0199421.s002.docx]

S1 File. Characteristics of Studies Included in the Meta-Analysis (*k* = 63).

| Study | Setting | Population Age  Characteristics  (in years) | Intervention Description | Study Design | *N* (Baseline) | Participant Selection | Outcomes used in meta-analysis |
| --- | --- | --- | --- | --- | --- | --- | --- |
| Abel 2008 [41] | United States (city non reported) | *Mean* = 11.39 (*SD* = 0.83) | A program to promote healthy family functioning, and increase the awareness of sexual abstinence as a positive choice for youth. Content focuses on asset building, parent-teen communication and relationships, self-esteem, healthy relationships, dealing with peer pressure, and the benefits of sexual abstinence. | Before-after study | *N* = 130 | Non-random | Abstinence self-efficacy  Intention to refuse sex |
| Achiong-Alemañy 2011 [42] | Cuba (Province of Matanzas) | Range: 15-16 | Educational intervention in HIV / AIDS (intervention not described). | Before-after study | *N* = 420 | Non-random | Knowledge  Attitudes  Condom use |
| Ajuwon 2008 [43] | Nigeria (Oyo State) | Adolescent peer educators (secondary school students) | An AIDS prevention intervention for primary health care workers. It includes a module on the overview of the HIV/AIDS situation in Oyo State and in Nigeria. Contents on the socio-cultural sexual risk practices in the area, the relationship between STIs and HIV; HIV prevention strategies, and a group work for the development of work-plans for AIDS prevention. | Before-after study | *N* = 2,082 persons (of them 48.2% were adolescents) | Not reported | Knowledge |
| Aramburú 2012 [44] | Panama (Panama City) | *Mean* = 15 (*SD* is not provided) | Educational impact of peer-intervention to improve knowledge and attitudes towards HIV/AIDS. Intervention was divided in four modules: theatrical representations, group dynamics, lectures and videos. | Before-after study (includes control group) | *N* = 652 (I = 349; C = 303) | Non-random | Knowledge |
| Armitage 2010 [45] | England (city not reported) | Range: 16-18 | Intervention consisted of three sections, aims at changing attitude, perceived behavioral control and subjective norm. | Randomized individual trial | *N* = 288 (I = 141; C = 138) | Random | Attitudes  Subjective norms  Self-efficacy to use condoms |
| Atwood 2012 [46] | Liberia (Monrovia) | *Mean* = 16.3 (*SD* = 1.7) | A program to promote positive condom attitudes, increase skills and self-efficacy to refuse sex, negotiate condom use, and use condoms effectively. | Group randomized controlled trial | *N* = 812  (I = 400; C = 412) | Randomized group trial | Knowledge  Attitudes  Subjective norms  Self-efficacy to use condoms  Abstinence self-efficacy |
| Barker 2012 [47] | United States  (two midsized cities in the  northeastern and two midsized cities in the  southeastern) | Range: 13-18 | Eight 90-min sessions implemented in two days. The intervention aims to prevent HIV behaviors, provides skill-based instruction, decision-making and communication. It includes group discussions, arts and crafts, role plays, didactics, story-telling, and videos. | Randomized individual trial | *N* = 1,654 (I = 821; C = 833) | Random | Knowledge |
| Bartlett 2010 [48] | United States  (city not reported)  (Rural county in the southeastern) | Range: 11-14 (*Mean* = 12.9) | An intervention that includes communication skills, healthy relationships, assertiveness, human sexuality, risky behaviors related to contracting HIV infection, and self-protection against HIV infection. A woman affected by HIV participates as a guest speaker at the end of the session. | Before-after study | *N* = 10 | Random | Knowledge  Self-efficacy to use condoms |
| Baumler 2012 [49] | United States  (Houston) | *Mean* = 12.4 (*SD* = 0.59) | 50-minute 24 lessons intervention that included information about puberty, reproduction, and STIs, and setting personal limits and practicing refusal skills related to sexual behavior, characteristics of healthy dating relationships, the importance of HIV, STI, and pregnancy testing. | Group randomized trial | *N* = 817 (I = 308; C = 509) | Non-random | Knowledge  Attitudes  Subjective norms  Self-efficacy to use condoms  Abstinence self-efficacy |
| Berglas 2016 [50] | United States  (Los Angeles) | Range: 14-15 | An intervention to improve sexual health by reducing the risk of pregnancy and STIs and improving adolescent’ ability to manage their sexuality respectfully. Components are classroom curriculum, parent education, peer advocate program, and sexual health services. | Group randomized trial | *N* = 741 ( I = 412; C = 329) | Random | Condom use |
| Birch 2011 [51] | United States  (Georgia and Virginia) | Range: 12-15 | Sexual education program focused on abstinence. The curriculum is implemented in school settings during health classes. | Group randomized trial | *N* = 1,628 (I = 648; C = 980) | Random | Attitudes  Self-efficacy to use condoms  Condom use intention |
| Cai 2008 [52] | China (Shanghai) | *Mean* = 17.22 (*SD* = 0.89) | Peer-led HIV/AIDS prevention to increase knowledge about HIV transmission, to improve behavior and behavioral intention to protect adolescents from HIV/AIDS; and to eliminate prejudice and stigmatization towards people living with HIV/AIDS. | Randomized individual trial | *N* = 1,630 (I = 968; C = 982) | Random | Knowledge  Condom use intention |
| Chong 2013 [53] | Colombia (city not reported) | Range: 15-16 | An online comprehensive curriculum aimed at shaping adolescents’ understanding and perceptions of sexuality, risks, reproductive health, sexual rights and dating violence. All modules have a human rights approach to pregnancy and sexuality. Contents are focused on the right to say no to sex, to access basic health services, to access family planning services, and to live without sexual violence. | Group randomized trial | *N* = 4,599 (I = 3,122; C = 1,477) | Non-random | Knowledge  Attitudes |
| Claramunt-Busó 2011 [54] | Spain (Valencia) | Range: 12-17 (*Mean* = 14.4; *SD* = 1.19) | An affective-sexual program aimed at increasing basic knowledge about human sexuality, developing a critical sense that allows avoiding myths and unfounded fears; promote an ethical attitude towards sexuality. It promotes communication on respect and responsibility in personal relationships and tolerance towards different expressions of sexuality. The intervention also aims at developing self-esteem through one's own sexual identity and acquiring the skills necessary to live sexuality | Non-randomized group trial | *N* = 513 (I = 321; C = 192) | Non-random | Knowledge  Attitudes |
| Coffman 2011 [55] | South Africa (Cape Town) | *Mean* = 14; *SD* =.86 | Curriculum aimed at reducing the transmission of HIV/AIDS and other STIs, reducing drug and alcohol abuse, and increasing positive use and experience of free and leisure time. | Group randomized trial | *N* = 2,429 (Ns by study group not  reported) | Random | Self-efficacy to use condoms |
| Constantine, 2015 [56] | United States  (Los Angeles) | Range: 12-18 | Curriculum aimed at reducing risk of pregnancy and STIs, improving ability to manage sexuality. It adopts a rights-based framework that focuses on human rights, gender equality, access to health care services, and critical thinking, and emphasized the relationship between broader social and cultural factors and individuals’ sexual decisions. | Group randomized trial | *N* = 1,750 (I = 934; C = 816) | Non-random | Knowledge  Attitudes  Self-efficacy to use condoms  Communication about sex with the sexual partner  Condom use intention |
| Cornelius 2013 [57] | United States (city not reported) | Range: 13-18 (*Mean* = 15.4; *SD* = 1.7) | Program aimed at providing information on understanding HIV, sexual decision making, developing condom skills, learning and practicing assertiveness communication skills, personalizing risks. It includes booster’s strategies (text messages, pictures, and videos). | Before-after study | *N* = 40 | Mixed | Knowledge  Attitudes  Condom use |
| Coyle 2013 [58] | United States (northern California) | *Mean* = 16.2 | HIV/STI and pregnancy prevention program aimed at reducing unprotected sexual intercourse by increasing condom use and reducing sexual intercourse. | Group randomized trial | *N* =  (I = 220; C = 217) | Not reported | Knowledge  Attitudes  Subjective norms  Self-efficacy to use condoms  Abstinence self-efficacy  Communication about sex with the sexual partner  Condom use |
| Cronin 2014 [59] | United States  (Missouri) | Range: 11-19 | Comprehensive sex education program aimed at promoting safer sex and abstinence and providing adolescents the necessary tools to make responsible decisions about their own sexual behaviors. | Before-after study | *N* = 241 | Non-random | Knowledge  Condom use intention |
| Dinaj-Koci 2015 [60] | The Bahamas  (New Providence) | *Mean* = 14.5 years; *SD* = 0.7 | Sexual risk reduction intervention aimed at improving threat and coping appraisals of risk-related situations and increase HIV-related skills and knowledge. | Randomized individual trial | *N* = 941  BFOOY- no parents  (*N* = 389)  HFLE-no parents) (*N* = 552) | Non-random | Knowledge  Self-efficacy to use condoms  Condom use intention  Intention to refuse sex |
| Escribano 2015 [61] | Spain (Alicante, Asturias, Castellón, Granada, and Murcia) | *Mean* = 14.76; *SD* = 0.75 | Curriculum aimed at increasing the level of knowledge and attitudes related to STIs, and reducing sexual risk behavior. The components are transmission of information, social skills training, problem-solving training, and strategies to maintain safer sexual behavior. | Group randomized trial | *N* = 1121 (I = 622; C = 499) | Non-random | Knowledge  Attitudes  Subjective norms  Self-efficacy to use condoms |
| Espada 2012 [62] | Spain (Alicante, Asturias, Castellón, Madrid, and Murcia) | *Mean* = 15.73, *SD* = 0.72 | Program aimed at increasing the level of knowledge and attitudes related to HIV/AIDS, and reducing risky sexual behavior. The components are transmission of information, social skills training, problem-solving training, and strategies to maintain safer sexual behavior. | Group randomized trial | *N* = 827 (I = 607; C = 220) | Non-random | Knowledge  Attitudes  Subjective norms  Condom use intention |
| Fernández 2008 [63] | Puerto Rico (city not reported) | Range: 11–14 | Intervention to increase HIV/AIDS knowledge. Activities included group discussion, audiovisual aids, debates, brainstorming, patient testimony, reflection, and critical thinking. | Randomized individual trial | *N* = 173 (I = 91; C = 82) | Non-random | Knowledge |
| García 2009 [64] | Cuba (Pinar del Río) | Range (mostly): 18 - 19 | Educational intervention designed from the training of peer promoters with the aim of increasing the level of knowledge about HIV prevention. | Before-after study | *N* = 358 | Non-random | Knowledge |
| Grose 2014 [65] | South Africa (KwaZulu-Natal) | Range: 12.66 -15.48 | Program aimed at reducing pregnancy through delaying the initiation of sex, reducing the frequency of sex, increasing the use of contraception, increasing self-worth and self-respect, improving perceptions of peer norms and behaviors about sex, building communication skills, and addressing assumptions and beliefs about gender and sexuality. | Before-after study | *N* = 95 | Non-random | Knowledge  Attitudes  Self-efficacy to use condoms |
| Hadley 2016 [66] | United States  (Philadelphia and Providence) | Range: 13-18 (*Mean* = 15.46; *SD* = 1.64). | A family-based intervention aimed at improving parent-adolescent general and sexual communication, parental monitoring, and adolescent self-efficacy for engaging in HIV prevention behaviors (e.g., negotiating safer sex with partners, avoiding sexual situations, purchasing and using condoms). | Randomized individual trial | *N* = 170 (I = 83; C = 87) | Not reported | Knowledge  Self-efficacy to use condoms  Condom use |
| Haglund 2008 [67] | United States  (city not reported) | Range: 12 - 19 | An intervention as a booster session of a comprehensive sexuality education. Contents include sex and abstinence, strategies for choosing and maintaining abstinence, and suggestions for parents to help youths be abstinent. It includes six interactive sessions with discussion questions, case studies, and exercises. | Serial cross-sectional study | *N* = 33 | Non-random | Communication about sex with the sexual partner |
| Harrison 2016 [68] | South Africa  (KwaZulu-Natal) | Range: 14 -17 | Intervention aimed at increasing condom use and delaying sexual debut as prevention methods. Contents include gender-related attitudes and social norms, self-efficacy, communication, and negotiation skills, and knowledge, attitudes, and behaviors for HIV/AIDS and pregnancy prevention. | Non-randomized group trial | *N* = 933 (I = 628; C = 305) | Non-random | Knowledge  Self-efficacy to use condoms  Communication about sex with the sexual partner  Condom use |
| Herrman 2016 [69] | United States  (city not reported) | Range: 12-19 | Program aimed at preventing adolescent pregnancy by fostering relationship skills and a sense of responsibility related to masculinity, sexual activity, relationships, and parenting in adolescent males. It includes participatory lessons, case studies, roleplays, videos, games, and activities assist participants in self-reflection and personal growth. | Before-after study | *N* = 159 | Non-random | Attitudes  Subjective norms  Condom use |
| Hill 2008 [70] | England (East Anglia) | Range: 16-18 | 20-min health promotion intervention. It includes a leaflet, a quiz, and prize draw.  It is designed to have impact on attitudes, normative beliefs, self-efficacy, intention to use condoms and condom use. | Randomized individual trial | *N* = 567 (I = 285; C = 282) | Random | Attitudes  Subjective norms  Self-efficacy to use condoms  Condom use intention  Condom use |
| Jahanfar 2009 [71] | Malaysia (Ipoh,  Perak) | Range =15- 19  (*Mean* =17.65; *SD* = 0.73) | 2-hour talk intervention on sexual risk prevention. Contents included the definition of friendship and relationship, level of closeness between male and female friends, physical relationship, gauging participants’ knowledge on risk factors in sexual relationship, identifying the perception on HIV, STIs, and prevention of pregnancies and STIs. | Before-after study | *N* = 182 | Non-random | Knowledge |
| Jones 2013 [72] | Trinidad and Tobago | Range: 11-18 | HIV/AIDS educational intervention. Contents includes information about HIV, modes of transmission, risk factors, and effects of HIV/AIDS on the health of individuals, how to prevent the spread of HIV, information on assertiveness skills and how to say no to unwanted sex. | Group randomized trial | *N* = 196 (I = 92; C = 104) | Non-random | Knowledge  Attitudes  Self-efficacy to use condoms  Intention to refuse sex |
| Klein 2011 [73] | United States  (San Francisco Bay Area) | Range: 14 -18 | A social skills intervention aimed at reducing HIV sexual risk behaviors. Contents include HIV risk reduction knowledge and seek to enhance communication, condom use, and relationship skills through behavioral skills practice, group discussions, lectures, role-playing and take-home exercises. | Group randomized trial | *N* = 178  (I = 91; C = 87) | Not reported | Knowledge  Self-efficacy to use condoms  Condom use |
| Li 2011 [74] | China (Nanjing) | Range: 15 -19 | An abstinence-based intervention to delay the age of sexual onset. Contents included HIV-related perceptions, stigmatizing attitude toward people living with HIV/AIDS, intentions of health-related risk behaviors and sexual intercourse. Materials and activities related to condom use were removed from the original curriculum. | Group randomized trial | *N* = 304 (I = 140; C = 164) | Non-random | Knowledge  Attitudes |
| Lieberman 2012 [75] | United States  (Georgia) | I: *Mean* = 14.2: *SD* = 0.54;  C: *Mean* = 14.19; *SD* = 0.51 | Curriculum promotes abstinence from sexual activity until marriage to prevent pregnancy, STIs, and possible negative emotional consequences. Contents include the benefits and limitations of condoms in preventing pregnancy and STIs, in the context of promoting abstinence until marriage as the healthiest and most reliable choice. | Group randomized trial | *N* = 1,143 (I= 756; C = 387) | Non-random | Attitudes  Self-efficacy to use condoms  Intention to refuse sex |
| Mahat 2008 [76] | United States  (New Jersey City) | Range: 13 -  15 | Peer education program to improve the knowledge on sexual risk behaviors and change risk-taking behaviors. | Non-randomized group trial | *N* = 97 (I = 58; C = 39) | Non-random | Knowledge |
| Mahat 2010 [77] | United States  (Newark, New Jersey) | Range: 13 -15 years | Six 45-minute peer education implemented by peer educators. The intervention is aimed at promoting positive changes in risk behaviors in order to prevent infection with HIV and other sexually transmitted diseases. | Before-after study | *N* = 106 | Non-random | Knowledge  Self-efficacy to use condoms |
| Mahat 2016 [78] | United States (urban  School) | Range: 11 - 15 | HIV/AIDS Peer education program to increase knowledge about the virus. The program is implemented by peers previously trained. | Before-after study | *N* = 140 | Non-random | Knowledge |
| Malow 2009 [79] | United States  (Miami) | Range: 13 - 18 | A cognitive behavioral HIV risk reduction intervention that provides information to increase knowledge about HIV and the consequences of risky sexual behavior. It builds refusal skills and other communication skills with potential sexual partners. | Randomized individual trial | *N* = 246 (I = 116; C = 87) | Not reported | Knowledge  Attitudes  Self-efficacy to use condoms  Condom use intention |
| Markham 2014 [80] | United States  (urban and south-central area) | *Mean* = 12.6 (*SD* = .77) | Sexual risk reduction intervention aimed at promoting abstinence until older, responsibility, and self-respect. It included computerized skill-based activities to practice steps for correct condom use. | Group randomized trial | *N* = 2,342  (I = 1,069; C = 1,273) | Not reported | Knowledge  Attitudes  Self-efficacy to use condoms  Abstinence self-efficacy  Intention to refuse sex  Condom use |
| Mason-Jones 2011 [81] | South Africa (Western Cape) | Range: 15-16 | Program aimed at delaying sexual debut and to increase use of condoms for those who had already started to have sex. | Non-randomized group trial | *N* = 3872 (I = 1,868; C = 2,004) | Not reported | Condom use |
| Mathews 2016 [82] | South Africa (Western Cape) | Average: 13 | A multi-component intervention comprising an educational program, a school health service and a school safety program. Contents include assertive communication, gender power inequities, relationships, sexual decision-making, sexual violence, among others. | Group randomized trial | *N* = 3,284 (I = 1,650; C = 1,634) | Random | Knowledge  Attitudes  Subjective norms  Self-efficacy to use condoms  Self-efficacy to refuse sex  Condom use intention  Condom use |
| Morales 2014 [35] | Spain (Alicante, Asturias, Castellón, Madrid and Murcia) | Range: 15 - 18 (*Mean* = 15.72; *SD* = 0.73) | School-based HIV prevention intervention.  The intervention is composed of the following modules: information and cognitive restructuring; social skills training; problem solving training; maintenance strategies: self-management; and covert behavior rehearsal. | Group randomized trial | *N* = 832 (I = 241; C = 220) | Non-random | Knowledge  Attitudes  Subjective norms  Condom use intention |
| Morales 2015 [83] | Spain (Alicante, Asturias, Castellón, Granada, and Murcia) | Range: 14-16 | A program aimed at increasing knowledge about HIV and STIs, and perception of risk; to positively influence attitudes, norms and behaviours related to HIV risk reduction; and to improve problem-solving skills related to unprotected sex, and social skills to negotiate condom use. | Group randomized trial | *N* = 1,121 (I = 622; C = 499) | Non-random | Knowledge  Attitudes  Subjective norms  Condom use intention  Condom use |
| Morrison, Crean 2013 [84] | United States  (mid-size, northeastern U.S. city) | Range: 15 - 19 | A sexual risk reduction intervention that includes role-plays, discussion, interactive games and activities that targeted theoretical components of sexual risk reduction to prevent HIV transmission. | Randomized individual trial | *N* = 617 (I = 318; C = 299) | Not reported | Condom use |
| Morrison-Beedy, Jones 2013 [85] | United States (New York) | Range: 15-19 | Intervention addressed women’s concerns, such as how to persuade a partner to use a condom, obtaining condoms and how fertility could be jeopardized by risky sexual behavior. The structure and content of the intervention included strategies such as games, interactive group activities, and skits. Booster sessions at 3 and 6 months post-intervention are included. | Randomized individual trial | *N* = 639 (I = 329; C = 310) | Not reported | Condom use |
| Oman 2015 [86] | United States  (rural and urban settings) | Range: 11 - 14 | A school-based teenage pregnancy prevention program. It postulates that abstinence is most effective for preventing negative consequences of sexual activity, but it also provides instruction on contraceptive methods, including condoms, birth control pills, and the Depo-Provera shot, human growth and anatomy, reducing the possible negative effects of peer and media pressure on sexual decisions, and methods for refusing sexual approaches. | Non-randomized group trial | *N* = 6416 (I = 3,244; C = 3172) | Non-random | Knowledge  Attitudes  Intention to refuse sex |
| Peskin 2015 [87] | United States  (southeast Texas) | Range: 14.3 (*SD* = 0.59) | A prevention program aimed at making adolescents more responsible in sex and building refusal skills. It also includes contents on healthy and unhealthy friendships and dating relationships; anatomy and reproduction; social, emotional, and physical consequences of sex; communication skills; Internet communication and safety; consequences of teen pregnancy and STIs; knowledge and skills for condom and contraception use; and condom negotiation. | Group randomized trial | *N* = 1,697 (I = 939; C = 758 ) | Not reported | Knowledge  Attitudes  Subjective norms  Self-efficacy to use condoms  Abstinence self-efficacy  Intention to refuse sex  Condom use intention  Intention to refuse sex |
| Pinkleton 2012 [88] | United States  (Washington state) | Range: 12-18 | An intervention aimed at reducing the number of unintended pregnancies and STIs by promoting sexual abstinence. Contents include the analysis on how advertisers use sex to sell products to young people lesson encouraged adolescent to explore underlying messages about sexuality in advertisments. It addressed STIs and decision-making training about becoming or remaining sexually active. At the last session, participants create their own media messages. | Non-randomized group trial | *N* = 922 (I = 655 ; C = 267) | Non-random | Knowledge  Attitudes  Subjective norms  Self-efficacy to use condoms |
| Radha 2012 [89] | India | Range: 15- 16 approx. (grade 9th and 10th) | A school AIDS education program. Contents include education on reproductive physiology, basic medical facts about HIV/AIDS, and life skills education. | Before-after study | *N* = 580 | Non-random | Knowledge  Attitudes |
| Raghupathy 2013 [90] | United States | Range: 14 - 19 | An intervention that includes video clips of male and female condom demonstrations, “Jeopardy” style quizzes on condom use, teaches how and where to purchase condoms through role-plays, compared video clips demonstrating various responses. Basic HIV/AIDS and testing information is provided. | Group randomized trial | *N* = 355 (I = 173; C =162 ) | Not reported | Intention to refuse sex |
| Reyna 2014 [91] | United States (Southern Arizona, Northern Texas, and Central New York) | Range: 14-19 | Multicomponent educational program aimed at reducing sexual risk. This is an adapting of the existing curriculum (Reducing the Risk, or RTR) using tenets of fuzzy-trace theory. The core of this theory is the distinction between two types of mental representations of information: gist (bottom-line meaning) and verbatim (literal facts). | Randomized individual trial | *N* = 502 (I = 291; C = 211 ) | Not reported | Knowledge  Attitudes  Subjective norms  Self-efficacy to use condoms  Abstinence self-efficacy  Condom use intention  Intention to refuse sex  Condom use |
| Rohrbach 2015 [92] | United States (South and East Los Angeles) | Range: 12-18 | A rights-based sexuality education curriculum that include sexual rights and gender roles in relationships and media messages. The program provides contents on sexual and reproductive anatomy, pregnancy, STIs and contraception. | Group randomized trial | *N* = 2,033 (I = 1,089 ; C = 944) | Not reported | Knowledge  Self-efficacy to use condoms  Communication about sex with the sexual partner  Condom use intention  Condom use |
| Santibanez 2013 [93] | México  (San Quintin) | *Mean* = 13.70 (*SD* = 1.10) | A peer education program to deliver a reproductive health program. Contents include male and female anatomy, family planning, STIs, (including HIV/AIDS), use of condoms, and discussion of advantages and disadvantages of an early sexual debut and smaller family size. | Non-randomized group trial | *N* = 162 (I = 70; C = 92) | Non-random | Knowledge  Attitudes  Condom use intention |
| Shegog 2014 [94] | Unites States (southeast Texas) | Range: 13 - 15 | Internet-based sexual health life-skills curriculum adapted from an existing effective sexual health curriculum. It includes interactive exercises, quizzes, animations, peer video, and fact sheets. Selected lesson activities are tailored on gender, self-reported sexual experience and intentions. | Before-after study | *N* = 22 | Non-random | Self-efficacy to use condoms |
| Shin 2010 [95] | South Korea (Wonju) | Range: 12-15 | A mentoring program for promotion of sexual health. It provides accurate sexual  Information, including female and male genital organs and the sexual changes of adolescence; STIs and contraceptive methods and teach the participants about methods for the participants to protect themselves from sexual violence. | Before-after study | *N* = 33 (I = 17 ; C = 16) | Non-random | Knowledge  Attitudes |
| Starling 2014 [96] | United States  (New Mexico) | Range: 15.4 (*SD* = .97) | A sex education program that includes five units: introduction and initial assessment, media literacy, relationship formation and maintenance, safe behavioral choices/influences, and decision-making. | Before-after study | *N* = 173 | Non-random | Attitudes  Subjective norms  Self-efficacy to use condoms  Condom use intention |
| Taylor 2014 [97] | South African (KwaZulu-Natal) | Range: 12.6 (*SD* = 2.9) | A program aimed at providing information, building attitudes, and encouraging intentions to prevent teenage pregnancy. It includes role-playing, group discussions, debates, and viewing of videos made especially for the discussions. | Group randomized trial | *N* = 818 (I = 432 ; C = 386 ) | Random | Attitudes  Condom use intention  Intention to refuse  Condom use |
| Thato 2008 [98] | Thailand (Bangkok) | Range: 13–18 | A culturally sensitive comprehensive sex education program. Contents include human sexuality, prevalence of premarital sexual behavior and its consequences. It also provides information facts about STIs, HIV/AIDS, and pregnancy, prevention strategies, how abstinence reduces sexual risk. Thai values toward sexual activities are incorporated in the intervention. It addresses peer pressure/norms, negotiation skills, contraception methods, problem-solving and supportive resources. The program includes group discussion, videos, games, role-play, demonstration, brainstorming, and skill building activities. | Non-randomized group trial | *N* = 261 (I = 150; C = 146 ) | Mixed  (schools were not randomly selected, but participating classrooms were) | Knowledge  Condom use intention  Intention to refuse sex |
| Ulloa 2011 [99] | Cuba  (La Sierpe) | Range: 15-16 approx. (10^th^ grade) | A STIs prevention intervention. The contents include an introduction to the educational program and application of the survey, explanation of what are STIs, symptomatology and mode of transmission, classification of STIs and how to recognize them, risky sexual behaviors, possible complications of STI, and forms of prevention and control. | Before-after study | *N* = 68 | Non-random | Knowledge |
| Weed 2008 [100] | United States (Virginia) | 12 years approx.  (7^th^ grade) | An abstinence education curriculum is based on the abstinence education initiative as a primary prevention/risk avoidance strategy that could target the full spectrum of STDs, pregnancy and emotional health concerns for adolescents. | Non-randomized group trial | *N* = 662 (I = 421; C = 241) | Non-random | Attitudes  Subjective norms  Condom use intention |
| Wolfe 2009 [101] | Canada  (southwestern Ontario) | Range: 14-15 | A curriculum that address personal safety and injury prevention; healthy growth and sexuality, and substance use and abuse. It includes lesson plans, video resources, role-play exercises, rubrics, and handouts. | Group randomized trial | *N* = 1,722 (I = 968 ; C = 754 ) | Not-reported | Condom use |
| Ybarra 2015 [102] | Uganda (Mbarara) | Range: 13-18 (*Mean* = 16.1; *SD* 1.4) | A curriculum aimed at increasing HIV preventive information, motivation, and behavioral skills. Contents include information about HIV, aspects of motivation by focusing on problem-solving and communication skills, motivations to have sex or to be abstinent, condom use skills, consistent condom use negotiation. The program addresses cultural issues related to HIV preventive behavior, including coercive relationships between youth and romantic relationships with adults. | Randomized individual trial | *N* = 366 (I = 173; C = 193) | Random | Knowledge  Attitudes  Subjective norms  Condom use intention |

*Note.* I = Intervention and C = Control/Comparison Group.
